# Alterations of Cerebral Extracellular Vesicle microRNA Profiling Potentially Disrupts Brain Homeostasis Following Myocardial Infarction

**DOI:** 10.3390/biom16060776

**Published:** 2026-05-26

**Authors:** Md Monowarul Islam, Shouyi Liang, Lijun Sun, Guoku Hu, Neha Dhyani, Lie Gao, Tara L. Rudebush, Xue Xu, Jinpeng Liu, Irving H. Zucker, Changhai Tian

**Affiliations:** 1 Department of Toxicology and Cancer Biology, University of Kentucky, Lexington, KY 40536, USA; monowar.masud@uky.edu (M.M.I.); lijun.sun@uky.edu (L.S.); xxu244@uky.edu (X.X.); 2 Department of Cancer Biostatistics, University of Kentucky, Lexington, KY 40536, USA; shouyi.liang@uky.edu (S.L.); jinpeng.liu@uky.edu (J.L.); 3 Department of Pharmacology and Experimental Neuroscience, University of Nebraska Medical Center, Omaha, NE 68198, USA; cdut531@163.com; 4 Department of Cellular and Integrative Physiology, University of Nebraska Medical Center, Omaha, NE 68198, USA; ndhyani@unmc.edu (N.D.); trudebush@unmc.edu (T.L.R.); izucker@unmc.edu (I.H.Z.); 5 Department of Anesthesiology, University of Nebraska Medical Center, Omaha, NE 68198, USA; lgao@unmc.edu

**Keywords:** heart failure, cognitive impairment, cerebral extracellular vesicle, oxidative stress, neuroinflammation

## Abstract

Cognitive impairment (CI) is prevalent among heart failure (HF) patients. Although the brain injury in HF is multifactorial, oxidative stress and neuroinflammation are common pathological features of neurological disorders and are increasingly recognized as key mechanisms underlying CI. Extracellular vesicles (EVs) are well-established mediators of biological signaling in myocardial function and are widely recognized for transporting a variety of microRNAs. However, whether myocardial injury alters the miRNA profiles of brain EVs, potentially contributing to cognitive impairment (CI) by disrupting brain homeostasis, remains poorly understood. Using a rodent myocardial infarction (MI) model, we isolated brain EVs and characterized their miRNA profiling by means of small RNA sequencing. Our results demonstrate that miRNA profiles in brain EVs vary with HF progression. Only three miRNAs were significantly changed at 3 weeks post-MI, whereas thirty-two miRNAs and sixty-five miRNAs demonstrated significant changes post-MI, showed significant alterations at 6 and 12 weeks post-MI, respectively. Bioinformatic analysis suggests that some miRNAs against oxidative stress and inflammation were downregulated in brain EVs at 6 and 12 weeks post-MI. Conversely, several miRNAs responsible for oxidative stress and neuroinflammation were significantly increased, which may be of cardiac origin following MI. Collectively, these findings suggest that cardiac EVs may contribute to miRNA alterations in brain EVs, potentially driving CI by disrupting brain homeostasis.

## 1. Introduction

Cardiovascular disease including heart failure (HF) remains the leading cause of mortality, substantially impacting the health and economic burden in the United States and worldwide [1]. Furthermore, clinical and experimental evidence suggest that 20–80% of patients with myocardial ischemia are affected by cognitive impairment (CI), which manifests as memory and global cognitive deficits [2,3]. However, the underlying mechanisms by which ischemic myocardial injury contributes to the progression of CI remain elusive.

CI has been clinically reported in patients with various cardiac diseases including hypertension, atrial fibrillation and coronary artery diseases [2]. It is well-documented that cerebral blood flow (CBF) decline with increasing age and systemic inflammation has been implicated in the occurrence of CI among HF patients [4,5,6]. Gradual disruptions in cerebral hemodynamics appear to play a critical role in the pathogenesis of CI [7,8,9]. Although decreased CBF is a major contributor, CI has also been observed in the progression of HF, regardless of CBF impairment [10,11,12]. This suggests that other mechanisms may contribute to CI in response to myocardial injury. Extracellular vesicles (EVs) have been increasingly recognized as mediators for intra- and inter-organ communication in human diseases [13,14,15]. Some bioactive materials, including lipids, proteins and nucleotides such as non-coding RNAs, are abundant in EVs and play important roles in the pathogenesis of human diseases, including HF [16,17,18]. Recent studies from our laboratory and others have demonstrated that cardiac EV-mediated heart–brain axis crosstalk contributes to brain structural damage [19], oxidative stress [20] and neuroinflammation [21,22]. Cardiac EV-enriched miRNAs play critical roles in these pathophysiological processes in the brain following myocardial infarction (MI) [19,20,21]. Given that cardiac-derived miRNAs are potentially involved in disrupting brain homeostasis in HF, it remains unclear whether the miRNA expression profiling of cerebral EVs varies during the progression of HF, and if cerebral EV-enriched miRNAs are of cardiac origin and responsible for cerebral oxidative stress and neuroinflammation causing CI.

Here, we hypothesize that cardiac EVs are abundant with miRNAs which are dysregulated following myocardial infarction (MI) and circulate to the brain where miRNAs regulate brain oxidative stress and neuroinflammation, contributing to CI. To address this hypothesis, we investigated changes in miRNA profiles in brain tissue-isolated EVs following the progression of HF using a rat model. We analyzed the relationship between dysregulated miRNAs of brain EVs at different post-MI time points and cerebral pathological changes including oxidative stress, neuroinflammation and neuronal damage.

## 2. Materials and Methods

### 2.1. Rat Model of Heart Failure

Male Sprague–Dawley rats (180–200 g) were used to generate a chronic HF model by the ligation of the left anterior descending coronary artery. As previously described [23], the left anterior descending coronary artery was ligated following a left thoracotomy under isoflurane anesthesia (2%), and sham animals underwent a similar surgical procedure without coronary artery ligation. Long-acting Buprenorphine ER (1.2 mg/kg, sc) was used as analgesia during recovery from surgery. All experimental rats were subjected to echocardiography to determine cardiac function using a Vevo 3100 Imaging System with a 40 MHz probe (Visual Sonics, Inc. Toronto, ON, Canada) at 3 weeks, 6 weeks, and 12 weeks post-myocardial infarction (MI), respectively [20]. In this study, we targeted HFrEF (Heart Failure with reduced Ejection Fraction) in which an EF less than 45% was considered typical heart failure. Normal EF for rats ranges between 55 and 70%. Thus, MI animals with EF more than 45% were excluded from further study. At the end of the study for each animal, hemodynamic measurements were made using a Millar pressure catheter (AD Instruments, Colorado Springs, CO, USA) inserted into the left ventricle through the right carotid artery under 2% isoflurane anesthesia. All animal experimental protocols were approved by the Institutional Animal Use and Care Committee (IACUC) at the University of Nebraska Medical Center and the University of Kentucky and were carried out in accordance with the NIH Guidelines for the Care and Use of Laboratory Animals and with the ARRIVE guidelines.

### 2.2. EV Isolation

Brain tissue EV isolation. Brain EVs were isolated from sham and HF rats as described previously [24,25] with modifications. Briefly, hemi-brain tissues were minced and treated with 20 units/mL papain in Hibernate A solution (ThermoFisher Scientific, Waltham, MA, USA) at 37 °C in a shaker for 15 min, and then gently homogenized in cold Hibernate A solution. The brain homogenates were filtered through a 40 µm cell strainer and then followed by sequential centrifugations (300× *g* for 30 min at 4 °C; 2000× *g* for 30 min at 4 °C, and 10,000× *g* for 30 min at 4 °C) to remove cells, membranes and debris. The supernatants were passed through a 0.22 μm syringe filter, and then ultracentrifuged at 100,000× *g* for 90 min at 4 °C to pellet EVs. Isolated EVs were stored at −80 °C.

Cardiac EV isolation. Myocardial EV isolation was performed as previously described [26,27] with some modifications. Briefly, the heart was transcardially perfused with phosphate-buffered saline (PBS). The non-infarcted area of the left ventricle including border-zone and remote myocardial regions was minced (30 s, 4 °C) using fine sterile scissors (2 cm straight blade) in pre-cold PBS (100 μL per 100 mg tissue), followed by digestion in 0.1% type II collagenase (Sigma-Aldrich, St. Louis, MO, USA) at 37 °C for 30 min. The digested tissue was centrifuged twice at 400× *g* for 15 min to remove the tissues and cells. The supernatant was filtered through a 0.45 μm sterile filter and then subjected to ultracentrifugation at 100,000× *g* for 90 min at 4 °C to pellet the EVs (Optima L-100XP Ultracentrifuge, Beckman Coulter, Nicholasville, KY, USA). The resulting EV pellets were obtained and resuspended in 100 μL PBS.

Plasma EV isolation. Total EVs from serum were isolated using a total EV isolation reagent (Cat. 4478360, Invitrogen, Carlsbad, CA, USA) according to the manufacturer’s instructions.

### 2.3. RNA Extraction of Brain EVs and miRNA Sequencing

Total RNA including miRNA was extracted from brain tissue-derived EVs of sham and HF rats using miRNeasy (QIAGEN, Hilden, Germany). All extracted RNA was used in the library preparation following Illumina’s TruSeq-small-RNA-sample preparation protocols (Illumina, San Diego, CA, USA). Quality control analysis and quantification of the DNA library were performed using an Agilent Technologies 2100 Bioanalyzer High Sensitivity DNA Chip (Santa Clara, CA, USA). Single-end sequencing 50 bp was performed on Illumina’s HiSeq 2500 sequencing system following the manufacturer’s recommended protocols.

miRNA Sequencing and Bioinformatics analysis: Raw reads were processed and quantified using an in-house ACGT101-miR pipeline (LC Sciences, Houston, TX, USA). Briefly, adapter dimers, low-quality reads, low-complexity sequences, and reads corresponding to common RNA families (rRNA, tRNA, snRNA, snoRNA) and repetitive elements were removed. Clean reads with lengths between 18 and 26 nucleotides were aligned to species-specific precursor and mature miRNA sequences in miRBase v22.0 [28], allowing for one internal mismatch and length variation at the 3′ or 5′ ends. Only miRNAs matching the reference species were retained for quantification and downstream analysis.

Analysis of Differentially Expressed miRNAs: Differential expression analysis of miRNAs was performed using the DESeq2 package in R (version 1.38.0) [29]. Differentially expressed (DE) miRNAs were defined with an absolute log fold change |log2(FC)| > log2(1.5) and a false discovery rate FDR < 0.05.

The Prediction of Target Genes of miRNAs: Target genes of the DE miRNAs were identified using the multiMiR R package (version 1.20.0) [30], integrating miRTarBase (version 9) [31], TargetScan (version 8) [32], and miRDB (version 6) [33]. Functional enrichment analysis was performed on the identified target genes for each DE miRNA using the clusterProfiler package (version 4.6.0) [34], focusing on Gene Ontology (GO) biological process terms. GO terms with an FDR < 0.05 were considered statistically significant.

### 2.4. Quantitative Reverse Transcription Polymerase Chain Reaction (qRT-PCR)

Total RNAs were extracted and purified with TRIzol Reagent and miRNeasy Mini Kit (QIAGEN) from myocardial and plasma-derived EVs per the manufacturer’s recommendations. For miRNA analysis, a synthetic cel-miR-39 (Integrated DNA Technologies, Coralville, IA, USA) was pre-loaded to each denatured sample as a spike-in control to normalize the variation between samples during EV RNA extraction, and the reverse transcription of total RNA was performed using a TaqMan miRNA reverse transcription kit (Applied Biosystems, Foster City, CA, USA), per the manufacturer’s protocol. The relative expressions of miRNAs and cel-miR-39 were quantified by using TaqMan mature miRNA assays (Applied Biosystems) according to the manufacturer’s protocol and using the 2^(−∆∆Ct)^ method.

### 2.5. Statistical Analysis

All data are presented as mean ± standard error of the mean (±SEM). The normality of all data sets was assessed using the Kolmogorov–Smirnov and Shapiro–Wilk tests when the sample size was greater than 6 (*n* ≥ 6). A non-parametric test was carried out to compare two groups when the sample size was less than 6 (*n* < 6). Unpaired *t*-tests with Welch’s correction were used to compare data between two groups. One-way analysis of variance (ANOVA) was used for multiple comparisons using GraphPad Prism 9.2 software. Values of *p* < 0.05 were considered statistically significant.

## 3. Results

### 3.1. Myocardial Infarction Alters the miRNA PROFILING of Cerebral EVs

**Figure 1:**
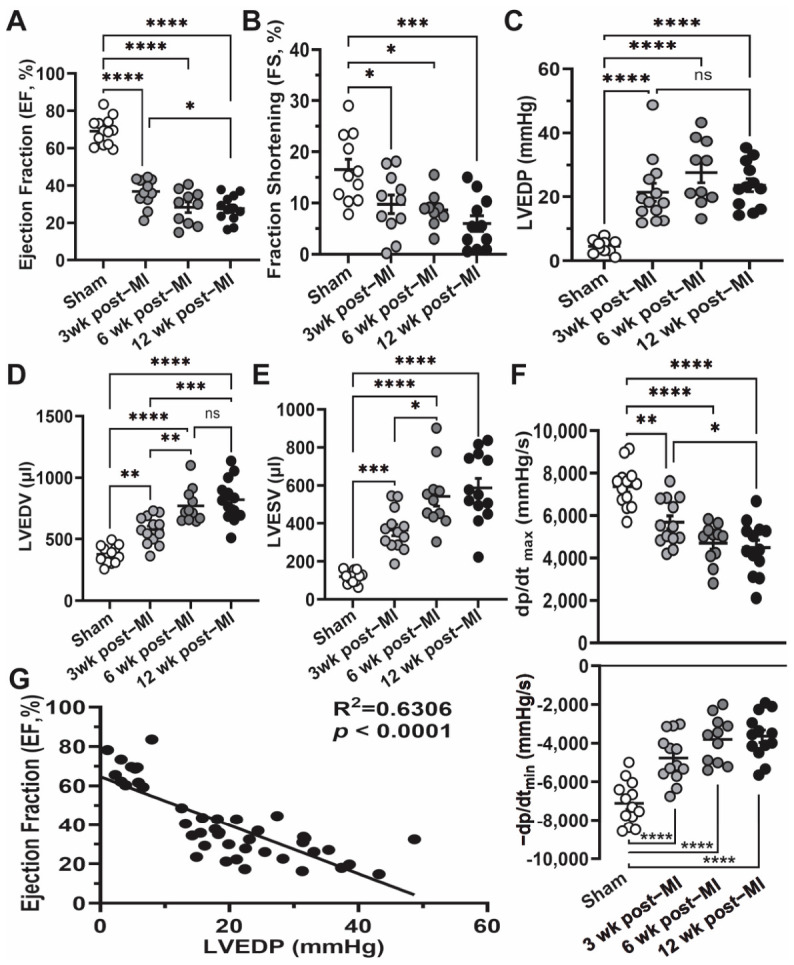
Echocardiographic and hemodynamic analyses demonstrate the heart failure phenotype. Echocardiographic and hemodynamic analyses were performed using sham and MI rats at 3 weeks, 6 weeks and 12 weeks post-MI, respectively. Ejection fraction (**A**), fraction shortening (**B**), left ventricular end diastolic pressure (LVEDP) (**C**), left ventricular end diastolic volume (LVEDV) (**D**), left ventricular end systolic volume (LVESV) (**E**) and dp/dt_max/min_ (**F**) were measured. The correlation between EF (%) and LVEDP (mmHg) was also analyzed (**G**). Tests were carried out for normality with the Kolmogorov–Smirnov tests, and *p* values were derived from Brown–Forsythe and Welch ANOVA tests (parametric test) (*n* = 10, ±SEM). * denotes *p* < 0.05, ** denotes *p* < 0.01, *** denotes *p* ≤ 0.001, **** denotes *p* < 0.0001, “ns” = not significant.

**Figure 2:**
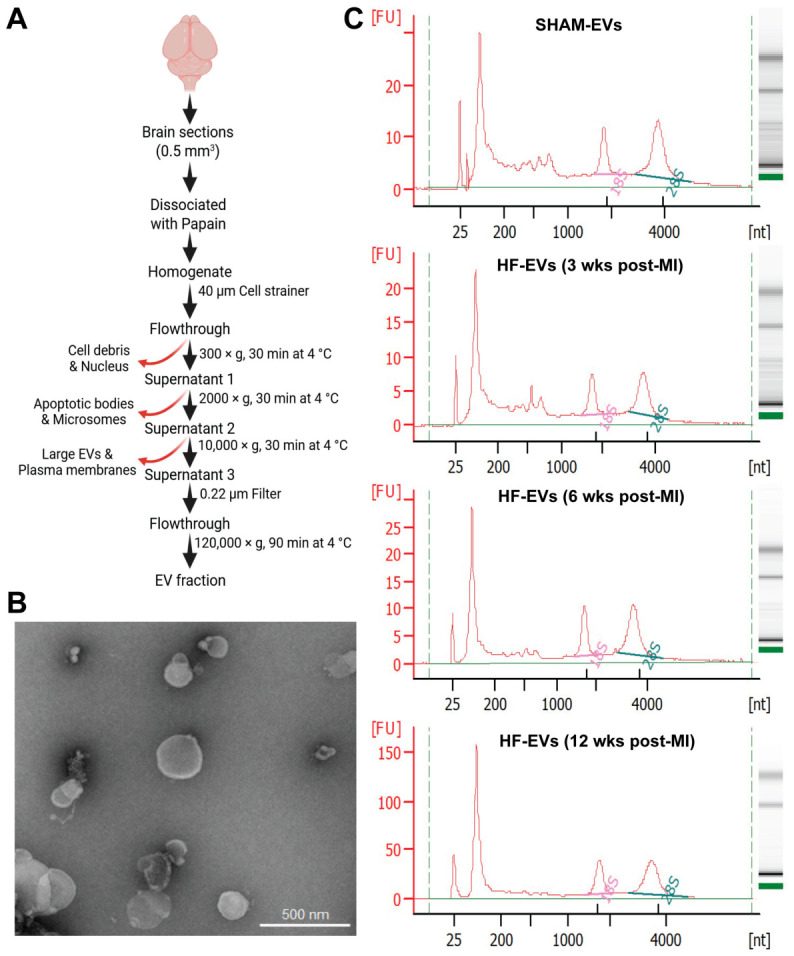
Pipelines for brain-isolated EVs and total RNA quality control of EVs. EVs were isolated from the hemi-brains of sham and MI rats, respectively, at 3 weeks, 6 weeks and 12 weeks post-MI following the illustrated procedures (red arrows indicate “removal”) (**A**); morphology of isolated brain EVs by TEM (**B**) and quality control of total RNAs (**C**). (*n* = 4/group).

Hemodynamic and echocardiographic evaluation of the post-MI HF model used here showed significant decreases in ejection fraction (EF%, Figure 1A) and fraction shortening (FS%, Figure 1B) in a time-dependent manner, beginning at 3 weeks post-MI, peaking at 6 weeks post-MI and stabilizing at 12 weeks post-MI. Other parameters of chronic HF including left-ventricular end-diastolic pressure (LVEDP, Figure 1C), left-ventricular end-diastolic volume (LVEDV, Figure 1D) and left-ventricular end-systolic volume (LVESV, Figure 1E) were all significantly increased at 3 weeks post-MI, peaked at 6 weeks post-MI and stabilized at 12 weeks post-MI. Consistently, left-ventricular maximum rate of pressure increase (dp/dt_max_) and decrease (dp/dt_min_) exhibited significant differences in a time-dependent manner and then stabilized at 12 weeks post-MI (Figure 1F). As critical hemodynamic markers in heart failure, these experimental rats exhibit the highly negative correlation between LVEDP and EF (%) (Figure 1G). Based on these echocardiographic and hemodynamic analyses, we selected four individual brains with typical HF phenotypes as biological repeats (Supplemental Table S1) from each group (sham, 3 weeks, 6 weeks and 12 weeks post-MI) for brain EV isolation following the outline illustrated in Figure 2A. Transmission Electron Microscopy (TEM) shows the typical cup-shaped morphologies of brain-isolated EVs (Figure 2B), and quality control of total RNAs extracted from brain EVs of each group suggest that all RNAs consist of fragments ranging from 20 to 200 nucleotides in addition to the 18S and 28S nucleotide fragments (Figure 2C), confirming their suitability for miRNA sequencing.

**Figure 3:**
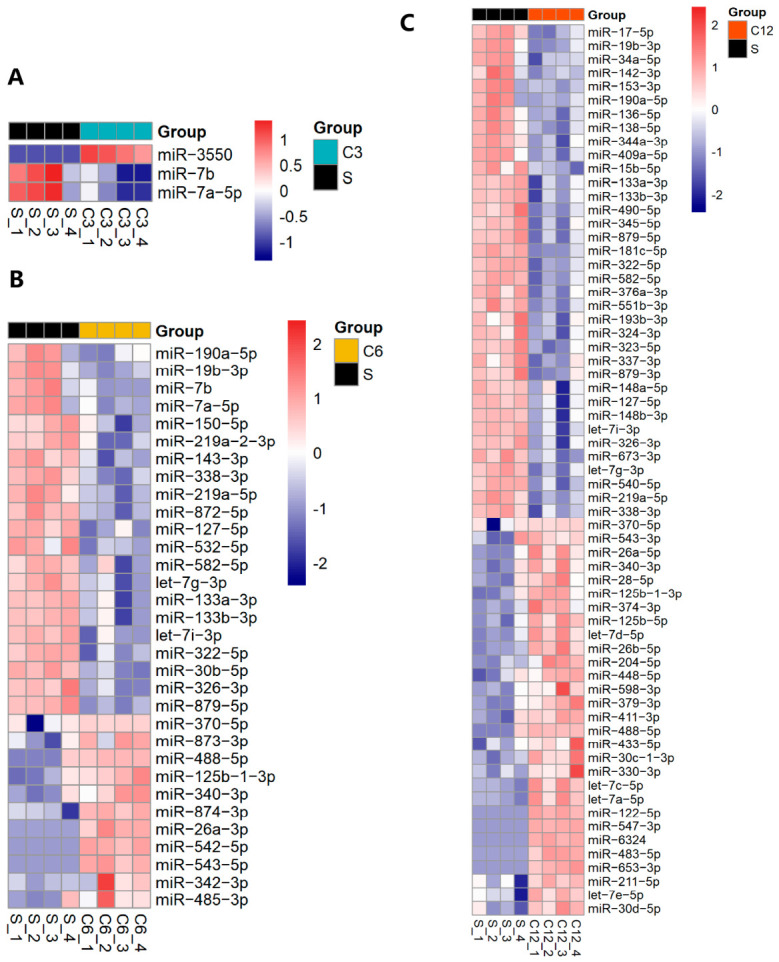
Differentially expressed miRNAs at 3, 6, and 12 weeks post-MI compared with sham-operated controls. Heatmaps show the top differentially expressed miRNAs ranked by fold change from four biological samples at each time point: (**A**) sham vs. 3 weeks post-MI; (**B**) sham vs. 6 weeks post-MI; (**C**) sham vs. 12 weeks post-MI (*n*= 4/each group). Upregulated and downregulated miRNAs are represented by red and blue colors, respectively. The thresholds of an absolute log fold change |log2(FC)| > log2(1.5) and a false discovery rate FDR < 0.05 were universally applied.

We extracted miRNA data from sham EVs and HF-EVs at different post-MI time points for analysis. Differential expression analysis of miRNAs between sham EVs and HF-EVs was determined using a threshold of |log2 fold change| > 0.585 and FDR < 0.05. Two miRNAs including miR-7b and miR-7a-5p were significantly decreased, whereas only one miRNA, miR-3550, was in higher abundance in cerebral EVs at 3 weeks post-MI compared to the sham group (Figure 3A). However, the number of dysregulated miRNAs enriched in the EVs isolated from the brain tissue increased relative to the progression of HF. A total of 11 out of 32 dysregulated miRNAs were significantly increased in brain tissue EVs at 6 weeks post-MI. These included miR-125b, miR-543 and miR-342-3p (Figure 3B). Although all cardiac dysfunctional parameters were stabilized at 6 weeks post-MI, 29 out of 65 dysregulated miRNAs were still highly enriched in brain tissue EVs at 12 weeks post-MI compared to the sham group (Figure 3C).

### 3.2. Brain Tissue EV-Enriched miRNAs Are Involved in Central Pathological Alterations

**Figure 4:**
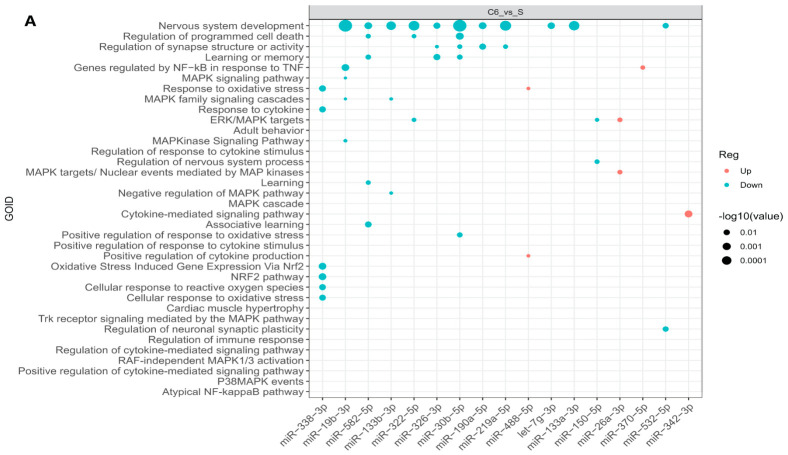

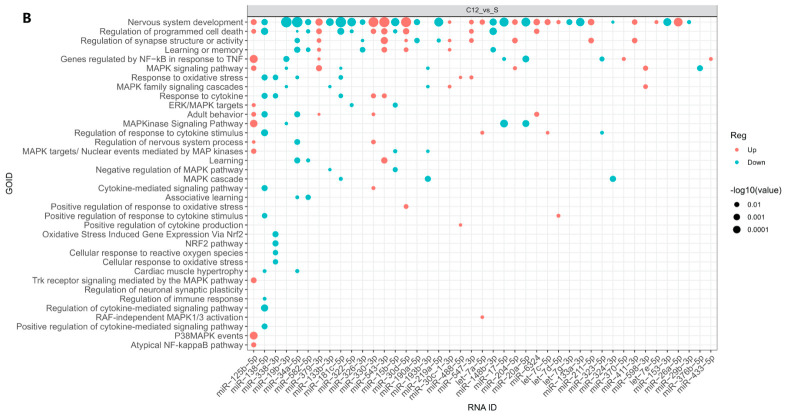
Gene Ontology (GO) enrichment analysis of the predicted target genes of differentially expressed miRNAs at 6 and 12 weeks post-MI compared with sham-operated controls. Bubble plots illustrate the enriched GO biological process terms of miRNA target genes, with the circle size corresponding to the enrichment significance −log_10_FDR and the circle color indicating the direction of miRNA regulation (red for upregulated and blue for downregulated). Panel (**A**) shows the enrichment results for 6 weeks post-MI vs. sham, and Panel (**B**) for 12 weeks post-MI vs. sham. Enriched GO terms with FDR < 0.05 were considered statistically significant.

To further confirm if these dysregulated miRNAs in brain tissue-isolated EVs contribute to the progression of cognitive impairment following the establishment of chronic HF, we performed Gene Ontology (GO) enrichment analysis for DE miRNAs at 6 weeks and 12 weeks post-MI, compared with sham controls. The bioinformatic analyses suggest that four out of 17 upregulated miRNAs in brain EVs at 6 weeks post-MI demonstrate positive correlation with oxidative stress and neuroinflammation, including miR-488-5p, miR-26a-3p, miR-370-5p and miR-342-3p; in contrast, 13 out of 17 downregulated miRNAs were negatively correlated with oxidative stress, neuroinflammation and neuronal dysfunctions. These included miR-338-3p, miR-582-5p and miR-30b-5p (Figure 4A). However, the number of miRNAs contributing to neuronal dysfunction, learning or memory impairment, oxidative stress and neuroinflammation increased in brain EVs at 12 weeks post-MI. Twenty out of 43 miRNAs enriched in brain tissue-isolated EVs, including miR-125b, miR-330-3p and miR-543-3p, contribute to these pathological alterations in chronic HF. The number of protective miRNAs potentially involved in these pathological alterations also increased but were significantly downregulated (Figure 4B). These included miR-138-5p, miR-34a-5p and miR-338-3p.

### 3.3. Brain Tissue EV-Enriched miRNAs Are Potentially of Cardiac Origin

**Figure 5:**
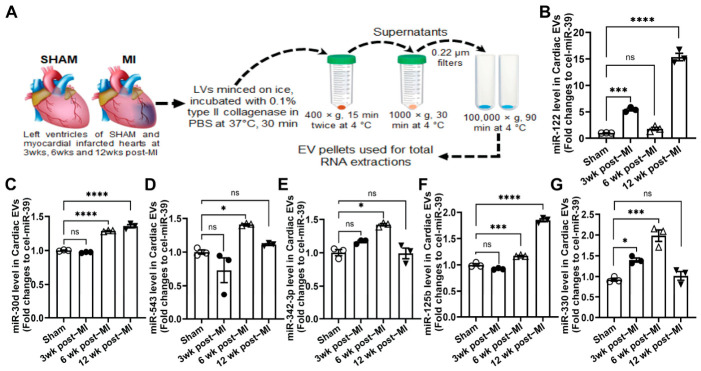
Cardiac miRNA profiling alterations following myocardial infarction. Cardiac EVs were isolated from the left ventricles of sham, 3 weeks, 6 weeks and 12 weeks post-MI rats, and then subjected to total RNA extraction, respectively, as illustrated (**A**); qRT-PCR analysis was performed with miRNA primers specific to miRNA-122 (**B**), miRNA-30d (**C**), miRNA-543 (**D**), miRNA-342-3p (**E**), miR-125b (**F**) and miRNA-330 (**G**). Cel-mir-39 was loaded as a spike-in control (*n* = 3, ±SEM). * denotes *p* < 0.05, *** denotes *p* < 0.001, **** denotes *p* < 0.0001 and “ns” = not significant.

To validate the potential cardiac origin of these upregulated miRNAs in brain tissue-isolated EVs, we isolated the cardiac EVs from the non-infarcted myocardium of left ventricles at 3, 6 and 12 weeks post-MI, alongside the corresponding areas in the sham-operated group as illustrated in Figure 5A. We then selectively detected several upregulated miRNAs in cerebral EVs and in cardiac EVs by means of qRT-PCR, including miR-125b, miR-543, miR-342-3p, miR-122, miR-30d and miR-330. The results demonstrated that miR-543, miR-342-3p and miR-125b levels were significantly upregulated in cardiac EVs at 6 weeks post-MI (Figure 5D–F), which is consistent with those observed in cerebral EVs at 6 weeks post-MI. Consistently, miR-122 and miR-30d levels significantly increased in cardiac EVs at 12 weeks post-MI (Figure 5B,C). However, although miR-330 was significantly increased in cerebral EVs at 12 weeks post-MI, this was not observed in cardiac EVs at 12 weeks post-MI but were significantly increased at 3 weeks and 6 weeks post-MI in a time-dependent manner (Figure 5G).

**Figure 6:**
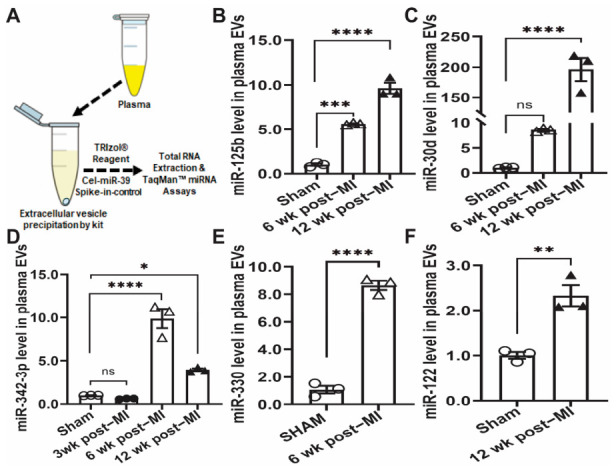
miRNA levels in plasma following myocardial infarction. Circulating EVs were isolated from plasma collected from sham and MI animals at 6 weeks and 12 weeks post-MI rats, respectively, as illustrated (**A**). qRT-PCR analyses show the miRNA-125b level (**B**) and miR-30d level (**C**) in plasma EVs of 6 weeks and 12 weeks post-MI, miRNA-342-3p level (**D**) and miRNA-330 level (**E**) in plasma EVs of 6 weeks post-MI, and miRNA-122 level (**F**) in plasma EVs of 12 weeks post-MI. Cel-mir-39 was used as a spike-in control (*n* = 3, ±SEM). * denotes *p* < 0.05; ** denotes *p* < 0.01, *** denotes *p* < 0.001, **** denotes *p* < 0.0001 and “ns” = not significant.

To further confirm the possibility that cerebral EV-enriched miRNAs may circulate from the heart following MI-induced HF, we collected circulating EVs from plasma samples as illustrated (Figure 6A). Interestingly, we observed that miR-125b was gradually upregulated in circulating EVs at 6 weeks and 12 weeks post-MI. This is consistent with the miR-125b level observed in both cardiac EVs and cerebral EVs following MI (Figure 6B). Moreover, miR-122 and miR-30d were also highly abundant in circulating EVs at 12 weeks post-MI, which were consistently observed in both cardiac EVs and cerebral EVs (Figure 6C,F). Furthermore, miR-342-3p level was highly enriched in circulating EVs at 6 weeks post-MI as observed in cardiac and cerebral EVs (Figure 6D). Although miR-330 was not altered in circulating EVs as observed in cerebral EVs at 12 weeks post-MI, it was significantly increased at 6 weeks post-MI (Figure 6E).

## 4. Discussion

Cognitive impairment (CI) and cardiogenic dementia are associated with a range of cardiovascular diseases including heart failure [2,12]. Studies show that 20–80% of patients with heart failure experience some form of cognitive decline, underscoring the high prevalence of CI [35,36,37]. However, the underlying mechanisms causing cognitive decline in heart failure patients remain to be elucidated. In the present study, we demonstrate that miRNA profiling in cerebral extracellular vesicles significantly changes with the progression of heart failure, and that the number of dysregulated miRNAs increases in a time-dependent manner. Moreover, bioinformatics analysis revealed that these dysregulated miRNAs of cerebral EVs at late stages of heart failure are highly associated with neuronal dysfunction, oxidative stress and neuroinflammation. Furthermore, the results of qRT-PCR show that the upregulated miRNAs in cerebral EVs isolated at different time points in the progression of HF are abundant in cardiac and circulating EVs, respectively. This suggests that cardiac EVs encapsulated with miRNAs may act as mediators that connect the heart and the brain in the progression of HF and eventually contribute to cardiogenic dementia in heart diseases. Our studies provide novel insights into the pathogenic mechanism underlying the inter-organ crosstalk between the heart and the brain and suggest new therapeutic strategies for mitigating brain lesions to prevent cognitive decline in the management and treatment of HF.

The pathophysiological mechanism of cardiogenic dementia is complex and characterized by reciprocal interactions between the heart and the brain, leading to brain structural changes, oxidative stress and neuroinflammation [19,21,22,38]. Emerging evidence suggests that subclinical changes in the cardiovascular system contribute to brain injury and cognitive vulnerability even in the absence of overt disease through several interconnected signaling pathways. These include neural [39], hormonal signaling [40,41], the renin–angiotensin system [42,43], systemic inflammation [44,45] and oxidative stress [46,47]. Our previous studies provide evidence that EVs derived from the injured heart also serve as novel carriers for delivering bioactive materials, especially miRNAs, and mediate heart–brain communication [20,21]. These findings underscore cardiac EVs as a pathogenic factor contributing to the development of CI following MI and the generation of chronic HF. Although cardiac EV-enriched miRNAs have been implicated in the disruption of brain homeostasis following MI [19,20,21], the dynamic alterations of cerebral EV-enriched miRNA profiles in the progression of HF remain elusive. Here, we used a rat MI model that exhibits typical HF pathological phenotypes, reaching stability at 12 weeks post-MI (Figure 1). Interestingly, we observed that three miRNAs were significantly dysregulated, but only one of the three was upregulated in cerebral EVs (Figure 3). It has been well-documented that both miR-7a-5p and miR-7b demonstrate neuroprotective roles in neurological disorders, such as Parkinson’s disease and Alzheimer’s disease [48,49,50]. However, they were significantly reduced in cerebral EVs at 3 weeks post-MI, suggesting that cardiac ischemia initially disrupts brain function at an early stage of HF. Although miR-3550 upregulation was observed in cerebral EVs, this miRNA is expressed only in rats and is positively associated with the development of diabetic nephropathy [51].

With the progression of HF, most pathological phenotypes of HF reached a peak at 6 weeks post-MI (Figure 1). On the other hand, the dysregulated miRNA profile of cerebral EVs was altered early following MI (Figure 3). It is well-known that miR-133a and miR-133b, also called muscle-specific miRNAs, are specifically expressed in cardiac and skeletal muscle and play protective roles in regulating oxidative stress and inflammation within the CNS following MI or in neurological disorders [52,53,54]. In addition to miR-133a and miR-133b, we also observed that the abundance of other miRNAs, such as miR-219-5p, miR-338-3p and miR-150-5p, is lower in cerebral EVs at 6 and 12 weeks post-MI compared to sham groups (Figure 3). Importantly, these miRNAs have been reported to have protective effects against oxidative stress and neuroinflammation [55,56,57]. In addition, some upregulated miRNAs were enriched in cerebral EVs following MI and have been shown to exert detrimental effects by promoting oxidative stress and neuroinflammation (Figure 4). For example, miR-125b-1-3p, which often functions alongside miR-125b, has been reported to act as a critical regulator of inflammation and oxidative stress [58,59]. Similarly, miR-122 upregulation has been found to promote pathological progression in neurological disease [60], cardiac [61] and hepatic disease [62,63], regulating oxidative stress and inflammatory pathways. These findings further confirm that cerebral EV-enriched dysregulated miRNAs contribute to the pathogenesis of CI following MI over time.

EVs have emerged as mediators of inter-organ communication in the pathogenesis of human diseases [14,64,65]. Our recent studies and others have demonstrated that the abundance of cardiac EVs has increased and that they mediate heart–brain communication following MI [20,21,22]. These studies and others also show that cardiac EV-enriched miRNAs not only elicit sympathetic excitation in brain stem nuclei by disrupting redox homeostasis but also cause structural damage by targeting hippocampal microtubules [19,20]. Here we profiled miRNA alterations in cerebral EVs following the progression of HF, and further validated the potential cardiac origin of these cerebral EV-enriched miRNAs (Figure 5 and Figure 6). These data support the hypothesis that cardiac EVs may contribute to the pathogenesis of cardiogenic dementia following MI by disrupting brain redox homeostasis and inducing neuroinflammation through the delivery of cardiac miRNAs. Additionally, surface receptors within the lipid bilayer of cardiac EVs, such as tetraspanins and integrins, may determine brain tropism by actively binding to target cells, including neurons and microglia [17,66]. While our proteomic data confirm the presence of Integrin β on cardiac EVs, the precise mechanism governing this post-MI brain tropism of cardiac EVs warrant further investigation. This study not only provides new insights into the mechanism by which the damaged heart regulates brain function, but also identifies promising therapeutic targets for HF treatment and management.

Limitations: Several limitations should be acknowledged. First, this study highlights only the importance of miRNAs enriched in cardiac-derived EVs in the pathogenesis of cardiogenic dementia, overlooking the potential roles of other bioactive components in both early and later stages of HF. Second, although cardiomyocytes account for 40% of the cardiac cellular population, the exact cellular origin of cardiac EV-enriched miRNAs remains to be investigated. Third, the specific downstream brain targets and functions of these upregulated miRNAs in cerebral EVs require further verification. Fourth, the study did not evaluate cognitive ability through post-MI behavioral testing, nor did it test potential treatment using antagomirs targeting these dysregulated miRNAs in HF.

## 5. Conclusions

The present study demonstrates that myocardial injury alters the miRNA expression profiles in brain EVs, with the extent of dysregulated miRNA profiles alongside HF progression. More importantly, bioinformatic analysis indicates that the levels of some protective miRNAs against oxidative stress and inflammation are significantly downregulated in brain EVs at 6 weeks post-MI. Conversely, miRNAs that promote oxidative stress and neuroinflammation are significantly upregulated following MI. By analyzing EVs isolated from the myocardium and the circulation, we identified the potential cardiac origin of several dysregulated brain EV miRNAs. These findings highlight the potential contributions of cardiac EV-enriched miRNAs to HF progression via the heart–brain axis where they disrupt CNS homeostasis and establish a potential causal link between cardiac dysfunction and CI.

## Data Availability

The datasets generated and analyzed in this study are publicly available in the NCBI BioProject database under the accession number PRJNA1452487 (https://www.ncbi.nlm.nih.gov/bioproject/PRJNA1452487), accessed on 22 May 2026.

## Supplementary Materials

The following supporting information can be downloaded at: https://www.mdpi.com/article/10.3390/biom16060776/s1, Table S1: Hemodynamic data and echocardiographic parameters of rats used for brain EV isolation.
