# Supplementary material for: Alterations of Cerebral Extracellular Vesicle microRNA Profiling Potentially Disrupts Brain Homeostasis Following Myocardial Infarction
Source: Biomolecules. 2026 May 26;16(6):776. doi: 10.3390/biom16060776 (PMC13297198; doi:10.3390/biom16060776)
Supplement: Supplementary file 1 [file biomolecules-16-00776-s001.zip › biomolecules-4328489-supplementary.pdf]

Table S1. Hemodynamic data and echocardiographic parameters of rats used for brain EV isolation.

	Samples	LVEDP	dp/dt_{max}	dp/dt_{min}	EF (%)	FS (%)	LVESV (μl)	LVEDV (μl)
**Sham**	S1	3.9	7620	-8476	60.3	7.8	122.9	310
	S2	1.1	6397	-7805	78.2	29	91.9	420.8
	S3	5.4	7495	-7746	68.7	23.3	123.1	392.7
	S4	2.3	7871	-7526	65.5	10.5	159	448
**3wks post-MI**	C3-1	21.1	6868	-5582	42.8	10.6	354.2	619.4
	C3-2	11.9	6838	-5707	43.2	11.4	262.8	462.8
	C3-3	27.4	7621	-5142	44.5	12.5	372.4	670.5
	C3-4	31.7	5037	-3136	33.1	0.18	384.6	574.9
**6wks post-MI**	C6-1	21.1	4233	-2916	22.3	7.4	553.8	712.6
	C6-2	18.3	4097	-3115	35.2	8.97	521.6	805.5
	C6-3	19.9	5112	-5007	30	6	452.2	645.9
	C6-4	38.6	5021	-5213	19.7	3	578.5	719.8
**12wks post-MI**	C12-1	17.8	5267	-4108	37.8	8.5	519.8	835.6
	C12-2	31.3	3115	-2942	16.4	6	732.4	875.7
	C12-3	22.4	3865	-3551	17.4	2.9	742.9	899.1
	C12-4	28.3	2114	-1913	22.6	5.5	816	1054.7

Note: MI, myocardial infarction; EF, ejection fraction; FS, fractional shortening; LVEDP, left ventricular end-diastolic pressure; LVEDV, left ventricular end-diastolic volume; LVESV, left ventricular end-systolic volume.
